# Prediction of prognosis in acute ischemic stroke after mechanical thrombectomy based on multimodal MRI radiomics and deep learning

**DOI:** 10.3389/fneur.2025.1587347

**Published:** 2025-04-30

**Authors:** Lei Pei, Xiaowei Han, Chenfeng Ni, Junli Ke

**Affiliations:** Department of Radiology, The Quzhou Affiliated Hospital of Wenzhou Medical University, Quzhou People’s Hospital, Quzhou, China

**Keywords:** multimodal MRI, radiomics, deep learning, acute ischemic stroke, prognosis

## Abstract

**Background:**

Acute ischemic stroke (AIS) is a major global health threat associated with high rates of disability and mortality, highlighting the need for early prognostic assessment to guide treatment. Currently, there are no reliable methods for the early prediction of poor prognosis in AIS, especially after mechanical thrombectomy. This study aimed to explore the value of radiomics and deep learning based on multimodal magnetic resonance imaging (MRI) in predicting poor prognosis in patients with AIS who underwent mechanical thrombectomy. This study aimed to provide a more accurate and comprehensive tool for stroke prognosis.

**Methods:**

This study retrospectively analyzed the clinical data and multimodal MRI images of patients with stroke at admission. Logistic regression was employed to identify the risk factors associated with poor prognosis and to construct a clinical model. Radiomics features of the stroke-affected regions were extracted from the patients’ baseline multimodal MRI images, and the optimal radiomics features were selected using a least absolute shrinkage and selection operator regression model combined with five-fold cross-validation. The radiomics score was calculated based on the feature weights, and machine learning techniques were applied using a logistic regression classifier to develop the radiomics model. In addition, a deep learning model was devised using ResNet101 and transfer learning. The clinical, radiomics, and deep learning models were integrated to establish a comprehensive multifactorial logistic regression model, termed the CRD (Clinic-Radiomics-Deep Learning) model. The performance of each model in predicting poor prognosis was assessed using receiver operating characteristic (ROC) curve analysis, with the optimal model visualized as a nomogram. A calibration curve was plotted to evaluate the accuracy of nomogram predictions.

**Results:**

A total of 222 patients with AIS were enrolled in this study in a 7:3 ratio, with 155 patients in the training cohort and 67 in the validation cohort. Statistical analysis of clinical data from the training and validation cohorts identified two independent risk factors for poor prognosis: the National Institutes of Health Stroke Scale score at admission and the occurrence of intracerebral hemorrhage. Of the 1,197 radiomic features, 16 were selected to develop the radiomics model. Area under the ROC curve (AUC) analysis of specific indicators demonstrated varying performances across methods and cohorts. In the training cohort, the clinical, radiomics, deep learning, and integrated CRD models achieved AUC values of 0.762, 0.755, 0.689, and 0.834, respectively. In the validation cohort, the clinical model exhibited an AUC of 0.874, the radiomics model achieved an AUC of 0.805, the deep learning model attained an AUC of 0.757, and the CRD model outperformed all models, with an AUC of 0.908. Calibration curves indicated that the CRD model showed exceptional consistency and accuracy in predicting poor prognosis in patients with AIS. Decision curve analysis revealed that the CRD model offered the highest net benefit compared with the clinical, radiomics, and deep learning models.

**Conclusion:**

The CRD model based on multimodal MRI demonstrated high diagnostic efficacy and reliability in predicting poor prognosis in patients with AIS who underwent mechanical thrombectomy. This model holds considerable potential for assisting clinicians with risk assessment and decision-making for patients experiencing ischemic stroke.

## 1 Introduction

Stroke, particularly acute ischemic stroke (AIS), is a major global health concern. It is not only one of the leading causes of death worldwide, responsible for approximately six million fatalities annually, but also the primary cause of mortality among residents of China (1). AIS accounts for 70% of all cerebrovascular diseases, primarily resulting from prolonged or permanent occlusion of cerebral vessels, which leads to ischemia and hypoxia in the brain tissue, causing localized neurological deficits or permanent loss of function (2). This condition is characterized by high rates of morbidity, disability, and mortality, with significant implications for patient prognosis, which is closely linked to the timeliness and efficacy of treatment. Despite substantial efforts by researchers worldwide to improve treatment approaches for AIS, including surgical and pharmacological interventions, the short-term prognosis remains unsatisfactory (3). The epidemiological features of AIS not only pose a severe threat to individual health and quality of life but also impose a substantial medical and economic burden on both society and families, emerging as one of the most pressing challenges in global public health.

The treatment of acute cerebral infarction is a complex multidisciplinary task that demands close collaboration across various departments and stages, with the ultimate goal of delivering timely and effective care to patients. Among therapeutic modalities, intravenous thrombolysis is widely employed, primarily through the administration of agents such as recombinant tissue plasminogen activator, urokinase, and tenecteplase, to restore blood flow (4). However, despite the ability of intravenous recombinant tissue plasminogen activator thrombolysis to alleviate symptoms in the short term in most patients, a subset of patients still face the risk of functional impairment and hemorrhagic transformation. In recent years, endovascular mechanical thrombectomy has emerged as a significant advancement in the treatment of AIS, particularly in patients with ischemic stroke due to large arterial occlusions, and it has been shown to substantially improve prognosis. However, some patients have a poor prognosis even after mechanical thrombectomy. Regardless of the treatment modality employed, early prognosis prediction for patients is of paramount importance, as it not only aids in the formulation of more precise pretreatment strategies but also facilitates the provision of more personalized care (5). Therefore, predicting the occurrence and progression of poor prognosis in AIS at an early stage and implementing proactive clinical interventions remain the central focus of current studies.

Previous studies have confirmed that factors such as the Alberta Stroke Program Early Computed Tomography (CT) Score (ASPECTS), patient age, presence of atrial fibrillation, and National Institutes of Health Stroke Scale (NIHSS) score are closely associated with the prognosis of recovery in patients with stroke (6). Smaller infarct volumes, well-developed collateral circulation, and lower NIHSS scores typically suggest a better prognosis for patients following endovascular treatment. Radiomics has recently emerged as a focal point of medical research and clinical practice. Advancements in neuroimaging have transcended its traditional role as a diagnostic tool and assumed an increasingly critical role in clinical decision-making (7). The integration of machine learning with radiomics has ushered in a revolutionary transformation in medical diagnostics, with successful applications in stroke research, such as the identification of acute cerebral infarction lesions based on CT- or magnetic resonance imaging (MRI)-derived radiomic features. Deep learning, a subset of machine learning techniques, constructs multilayered neural networks that can learn complex feature representations from vast datasets (8). Traditional stroke diagnostic methods have predominantly relied on physicians’ visual interpretation of brain images, whereas deep learning enables the automatic extraction of features from brain images, thereby assisting clinicians in making more accurate and timely diagnoses.

The field of medical diagnosis and treatment is currently faced with new opportunities and challenges arising from the integration of machine learning and radiomics. Currently, the application of MRI-based radiomics in predicting the prognosis of patients with stroke remains insufficient, with most studies relying solely on diffusion-weighted imaging (DWI) sequences, and the use of deep learning models is relatively limited. Considering this, the present study aimed to leverage multimodal MRI sequence data from patients with AIS, in conjunction with various machine learning algorithms and deep learning models, to construct a comprehensive predictive model for AIS prognosis after mechanical thrombectomy and assess its predictive performance. Through this study, we sought to provide a more accurate and holistic tool for the prognostic evaluation of patients with stroke.

## 2 Methods

### 2.1 Patients

**Figure 1:**
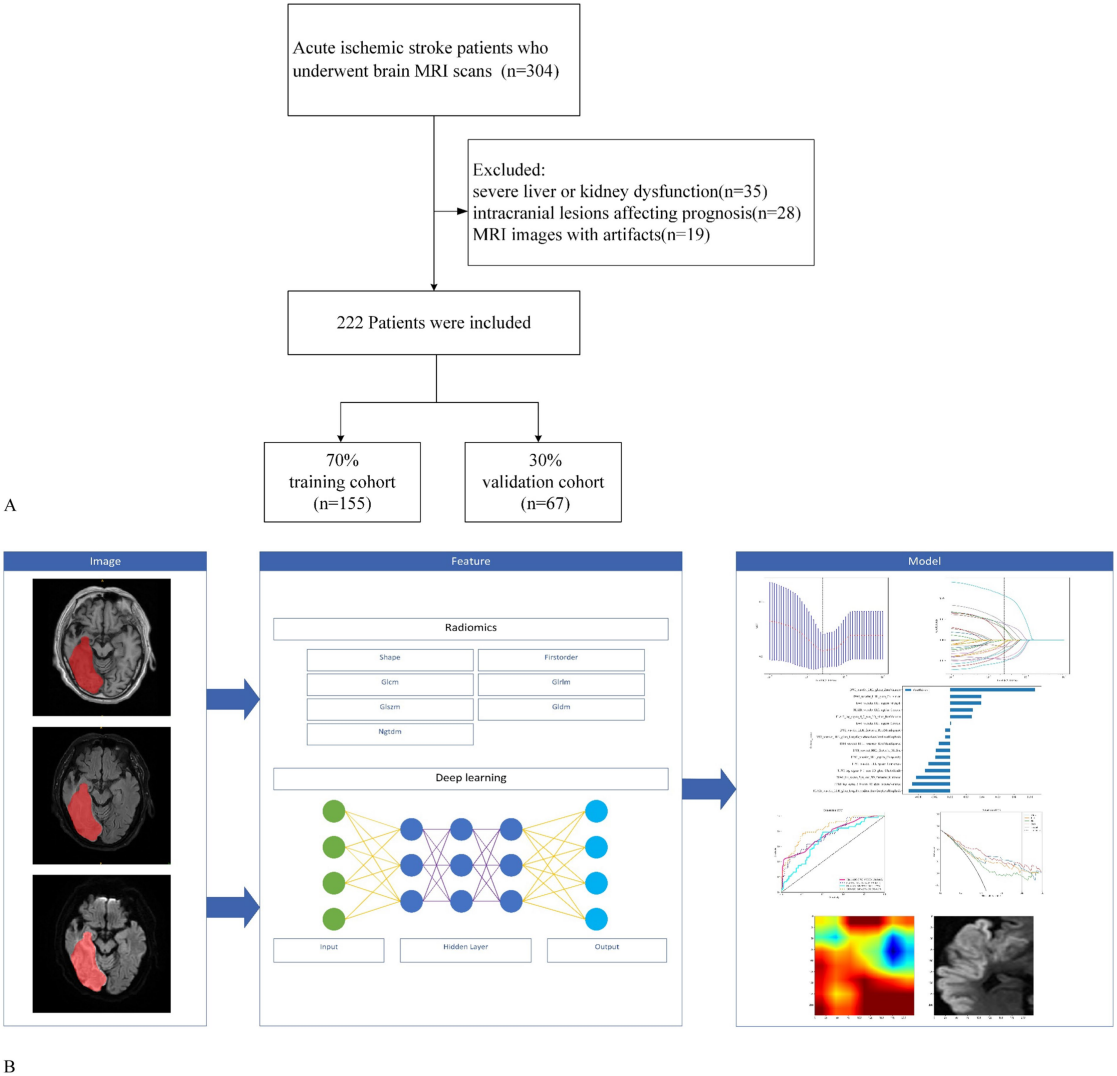
Workflow of the study. **(A)** Study flowchart of participant selection. **(B)** Workflow of the radiomics and deep learning analysis of AIS.

This retrospective study was approved by the Medical Ethics Committee of Quzhou People’s Hospital, which waived the requirement for informed consent from the participants. We included patients with AIS who underwent brain MRI at the hospital’s radiology department between January 2021 and May 2024. The diagnosis of AIS in this study strictly followed the current clinical guidelines, and all patients met the following criteria: (1) presence of symptoms of acute neurological deficit with an NIHSS score ≥2; (2) brain MRI-DWI sequence showing acute infarction in the responsible vascular blood supply area; and (3) exclusion of other non-vascular causes (such as epilepsy and metabolic encephalopathy). The inclusion criteria were as follows: (1) patients aged ≥18 years; (2) patients who met the diagnostic criteria for AIS; (3) patients who underwent high-quality MRI scans upon admission with complete clinical data; (4) patients who received mechanical thrombectomy treatment; and (5) patients who underwent MRI examinations before mechanical thrombectomy. The exclusion criteria were as follows: (1) severe liver or kidney dysfunction, hematological disorders, or malignant tumors; (2) intracranial lesions affecting prognosis, such as trauma or tumors; and (3) MRI images with artifacts or other factors that compromised image quality. We conducted a retrospective analysis of clinical data and biochemical results, including age, sex, smoking history, alcohol consumption, and history of hypertension, diabetes mellitus, and cardiovascular diseases. Prognostic evaluation at discharge was performed using the modified Rankin Scale (mRS), with a score of 3–6 indicating poor prognosis, and a score of 0–2 indicating good prognosis. All enrolled patients underwent ICH imaging evaluation prior to mechanical thrombectomy, and the diagnostic criteria were based on the following features: abnormal isohyperintense lesions on T1WI (excluding vascular artifacts) and hypointensity with peripheral hyperintense rings on FLAIR; chronic microbleeds are characterized by hypointense lesions. In this study, the clinical guidelines for mechanical thrombectomy were strictly followed, and patients with ICH (24 h < onset) or a significant mass effect (blood loss > 30 mL) in the acute phase were excluded as absolute contraindications. For patients with chronic phase microhemorrhage (cerebral microhemorrhage < 5 mm) or old hemorrhage, we have established a multidisciplinary decision-making process in which at least two neurointerventional physicians and one neuroimaging expert jointly evaluate the patient’s bleeding stability, lesion location, and vascular pathway relationship, and make a comprehensive judgment based on the patient’s NIHSS score and clinical indications to decide whether they should be included. A total of 222 patients were randomly divided into training and validation cohorts at a ratio of 7:3. In the training cohort, clinical features with statistically significant differences were selected using logistic regression, and a clinical model was developed. The workflow of this study is illustrated in Figure 1.

### 2.2 Image acquisition

Magnetic resonance imaging was performed using two distinct MRI machines (Siemens Skyra 3.0 T MRI from Germany and GE Signa Voyager 1.5 T MRI from the United States). The patient was placed in a supine position and continuous scanning was performed from the feet to the head, covering the range from the posterior fossa to the cranial vertex. Standard cranial MRI protocols encompassing axial T1WI, fluid-attenuated inversion recovery (FLAIR), and DWI sequences were employed. The repetition times for the 3.0 T MR were 2719/8600/2000 ms, with echo times of 9/106/57 ms. For the 1.5T MR, the repetition times were 488/8000/3543 ms and the echo times were 15/100/133 ms. For both MR scanners, the slice thickness was 5 mm, the field of view was 24 × 24 mm, and the matrix size was 512 × 512 pixels.

**Figure 2:**
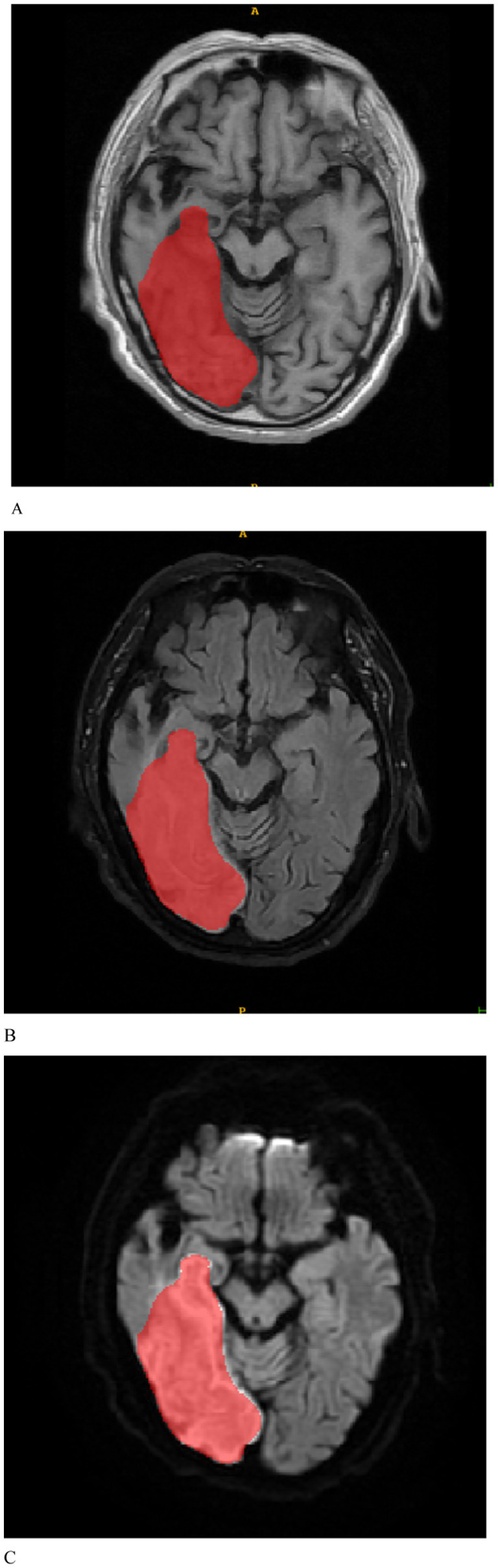
Based on the manually delineated regions of interest for patients with stroke, **(A–C)** represent the T1WI, FLAIR, and DWI sequences, respectively.

The MRI images were initially subjected to standardization procedures, including voxel resampling to 1 × 1 × 1 mm, adjustment of window width and level, N4 bias field correction, and normalization using *Z*-scores. Two radiologists, who were blinded to all patient information, assessed the MRI images. The axial MRI images of the enrolled patients were imported in DICOM format into the ITK-SNAP 3.8.0 software^1^. First, T1WI, FLAIR, and DWI sequences of the patients’ images were recorded. Given the challenges in delineating stroke lesion boundaries using T1WI and FLAIR images, stroke lesions were manually outlined on DWI images while considering the reference T1WI and FLAIR images (Figure 2). Disagreements were discussed until a consensus was reached. The software subsequently fused the region of interest for each image slice, yielding three-dimensional structural data of the lesions (volume of interest). To ensure the consistency and stability of lesion segmentation, 40 randomly selected MRI images from other patients were independently assessed by a second radiologist who applied the same methodology to outline the lesions and extract radiomics features. The intraclass correlation coefficient (ICC) was used to evaluate the consistency of the extracted features, with values exceeding 0.75 indicating good reproducibility.

### 2.3 Radiomics procedure

Radiomic features were extracted using the Pyradiomics package of Python 4.8.1, yielding 1,197 features for each region of interest. Feature type: the extracted image group features include multi-scale features after the original image features (Original), wavelet filtering (Wavelet) and LoG (Laplacian of Gaussian) filtering (*σ* = 2.0/3.0/4.0/5.0 mm), covering three categories of features: shape, first-order statistics, and texture (GLCM/GLRLM/GLSZM/NGTDM). The original image features were extracted directly after preprocessing (N4 bias correction and normalization), and the unfiltered feature set has been explicitly labeled as the “Original” group. To mitigate multicollinearity and achieve dimensionality reduction, the least absolute shrinkage and selection operator (LASSO) regression model was employed to select significant features. The optimal *λ* value corresponding to the minimum binomial deviance was determined using five-fold cross-validation, and features with non-zero coefficients were retained to form the final feature subset. The radiomics score was calculated based on the weighted summation of these features. A logistic regression classifier was employed for machine learning to construct the radiomic model, which was subsequently validated using an independent dataset. The radiomics score was computed using the following equation:


$$\mathrm{Radiomics score}=\sum \beta i\cdot \mathrm{Xi}+\mathrm{Intercept}\left(i=0,1,2,3\right)$$

Where *Xi* represents the radiomic feature values selected by LASSO, and *βi* is the coefficient corresponding to each selected feature *Xi*.

### 2.4 Deep learning procedure

In this study, we implemented a deep-learning network using the “PyTorch” framework in Python 4.8.1. By constructing a deep, non-linear convolutional neural network with multiple hidden layers, we progressively extracted and combined low-level features to form high-level abstract features, thereby simplifying the complex feature extraction process that is typical of traditional machine learning methods. We selected ResNet, a classical classification network known for its core residual structure, as the backbone model. By establishing shortcut connections between the earlier and later layers, ResNet effectively facilitates gradient backpropagation during training, thereby addressing the degradation problem that is inherent in traditional deep networks. ResNet101, consisting of 101 layers, is considered to have a relatively shallow structure. Building on ResNet101, we developed a 2.5D convolutional neural network (CNN) model using a residual structure to extract features. Using transfer learning, we converted the dataset features into vectors, which were then fused through fully connected layers to classify stroke prognosis.

In practice, we first identified the slice with the largest stroke area, assuming it to be the nth slice of the input volume. Subsequently, we extracted the (*n*−2), *n*, and (*n* + 2) slices for fusion, which were then input into the 2.5D CNN model. To enhance the generalizability of the model and mitigate overfitting, various data augmentation strategies were employed during training, including random translation, scaling, rotation, and shearing, as well as the addition of Gaussian noise, blurring, and Laplacian transformations. We applied L2 regularization to further optimize the model. The model parameters were optimized using the Adam algorithm, and all pretrained layers were fine-tuned to adapt to the current task. The initial learning rate was set to 0.0005, weight decay to 0.0001, and the L2 penalty coefficient to 0.01. Once the model achieved optimal accuracy on the test set, we saved all the model weights and validated the model using an independent test set.

### 2.5 CRD model establishment and statistical analysis

This study integrated clinical, radiomics, and deep learning models to construct a comprehensive multivariate logistic regression model, designated the CRD (Clinic-Radiomics-Deep Learning) model. A personalized nomogram was then generated to visualize the model, and decision curve analysis (DCA) was applied to quantify the net benefit across varying thresholds, thereby assessing the practical applicability of the CRD model. Clinical data were analyzed using Python 4.8.1 and SPSS version 26.0. For normally distributed data, the results are presented as mean ± standard deviation (*x* ± *s*) and were analyzed using independent *t*-tests. For non-normally distributed data, the median and interquartile range are reported, and comparisons were made using the Mann–Whitney *U* test. Categorical data are presented as frequencies (percentages) (*n* [%]), and comparisons were performed using chi-square tests. Logistic regression analysis was used to develop the predictive model, and the area under the receiver operating characteristic curve (AUC) was used to evaluate the predictive capability of the model. The DeLong test was employed to compare the AUCs of multiple models, DCA was used to evaluate the clinical utility of the model, and the optimal model was visualized as a nomogram. Calibration curves were used to assess the accuracy of the nomogram predictions, with statistical significance set at *p* < 0.05.

## 3 Results

### 3.1 Baseline characteristics

A total of 222 patients diagnosed with AIS were included in this study in a 7:3 ratio, with 155 patients in the training cohort and 67 in the validation cohort. Table 1 shows that there were no significant differences between the training and validation cohorts in terms of age, sex, NIHSS score at admission, hypertension, diabetes, cardiogenic diseases, smoking, drinking, or intracerebral hemorrhage (ICH) (*p* > 0.05). Table 2 indicates that the clinical data comparisons between the two cohorts were analyzed using independent *t*-tests or chi-square tests, with *p* < 0.05 considered statistically significant. Two independent risk factors for poor prognosis were identified: NIHSS score at admission and ICH.

**Table 1:** Patients’ baseline characters of our cohorts.

Characteristics	ALL	Validation cohort	Training cohort	*p*-value
Age	68.99 ± 12.05	70.39 ± 11.81	68.39 ± 12.14	0.324621
NIHSS at admission	7.41 ± 4.69	7.82 ± 5.49	7.23 ± 4.30	0.491425
Gender				1
0	98(44.14)	30(44.78)	68(43.87)	
1	124(55.86)	37(55.22)	87(56.13)	
Hypertension				0.448426
0	103(46.40)	28(41.79)	75(48.39)	
1	119(53.60)	39(58.21)	80(51.61)	
Diabetes				0.944928
0	168(75.68)	50(74.63)	118(76.13)	
1	54(24.32)	17(25.37)	37(23.87)	
Cardiogenic diseases				0.554017
0	198(89.19)	58(86.57)	140(90.32)	
1	24(10.81)	9(13.43)	15(9.68)	
Smoking				0.966679
0	147(66.22)	45(67.16)	102(65.81)	
1	75(33.78)	22(32.84)	53(34.19)	
Drinking				1
0	165(74.32)	50(74.63)	115(74.19)	
1	57(25.68)	17(25.37)	40(25.81)	
ICH				0.561503
0	201(90.54)	59(88.06)	142(91.61)	
1	21(9.46)	8(11.94)	13(8.39)	

**Table 2:** Comparison of patients’ baseline characters for poor prognosis in the training cohort and validation cohort.

Characteristics	Training cohort			Validation cohort		
	Good prognosis	Poor prognosis	*P*-value	Good prognosis	Poor prognosis	*p*-value
Age	67.29 ± 12.09	70.28 ± 12.10	0.139155	67.43 ± 11.63	76.86 ± 9.57	0.001881
NIHSS at admission	5.84 ± 3.08	9.63 ± 5.03	<0.001	5.52 ± 3.88	12.86 ± 5.19	<0.001
Gender			0.868411			0.266734
0	42(42.86)	26(45.61)		18(39.13)	12(57.14)	
1	56(57.14)	31(54.39)		28(60.87)	9(42.86)	
Hypertension			0.173769			0.224226
0	52(53.06)	23(40.35)		22(47.83)	6(28.57)	
1	46(46.94)	34(59.65)		24(52.17)	15(71.43)	
Diabetes			0.258194			0.478261
0	78(79.59)	40(70.18)		36(78.26)	14(66.67)	
1	20(20.41)	17(29.82)		10(21.74)	7(33.33)	
Cardiogenic diseases			1			0.804267
0	89(90.82)	51(89.47)		39(84.78)	19(90.48)	
1	9(9.18)	6(10.53)		7(15.22)	2(9.52)	
Smoking			0.484597			0.824463
0	62(63.27)	40(70.18)		30(65.22)	15(71.43)	
1	36(36.73)	17(29.82)		16(34.78)	6(28.57)	
Drinking			1			0.616132
0	73(74.49)	42(73.68)		33(71.74)	17(80.95)	
1	25(25.51)	15(26.32)		13(28.26)	4(19.05)	
ICH			0.004568			0.105598
0	95(96.94)	47(82.46)		43(93.48)	16(76.19)	
1	3(3.06)	10(17.54)		3(6.52)	5(23.81)	

### 3.2 Radiomics and deep learning models

**Figure 3:**
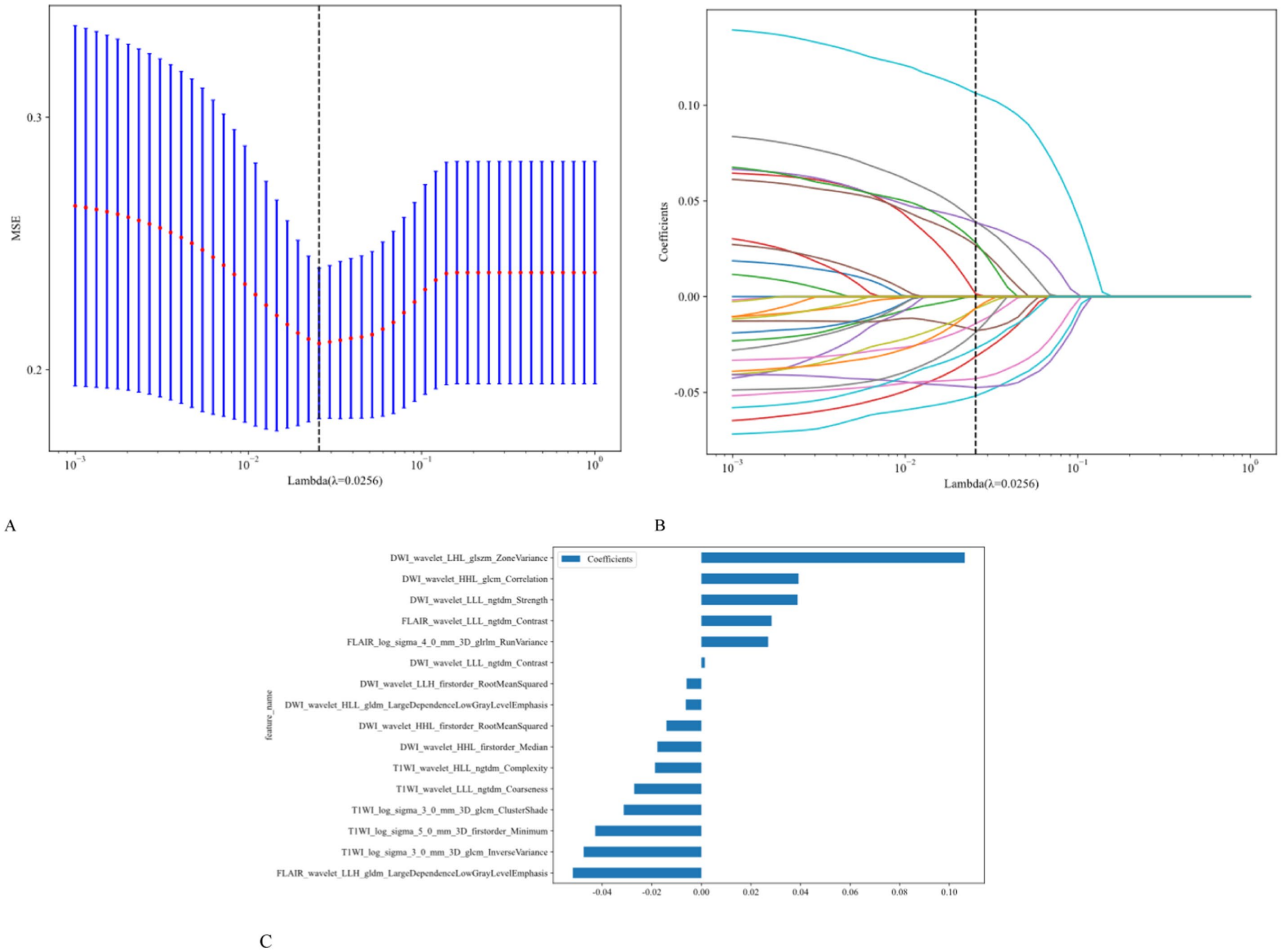
Utilization of the LASSO algorithm for feature selection. **(A)** The LASSO model employs five-fold cross-validation to select and fine-tune the parameters (*λ*). **(B)** Each colored line represents the coefficient of a specific feature, resulting in the final selection of 16 radiomic features **(C)**.

Using univariate logistic regression analysis and LASSO regression for dimensionality reduction, 16 of the 1,197 radiomic features were selected to construct the radiomics model. These features included 14 shapes, 234 first-order features, 286 features from the gray-level co-occurrence matrix (GLCM), 208 from the gray-level run length matrix (GLRLM), 208 from the gray-level size zone matrix, 182 from the gray-level dependence matrix, and 65 from the neighborhood gray tone difference matrix. The ICC was >0.75. Based on the LASSO regression model, the optimal *λ* obtained from five-fold cross-validation was used to select the best radiomic features with non-zero coefficients. The distribution of the LASSO coefficients for these features is shown in Figure 3.

### 3.3 CRD model

**Figure 4:**
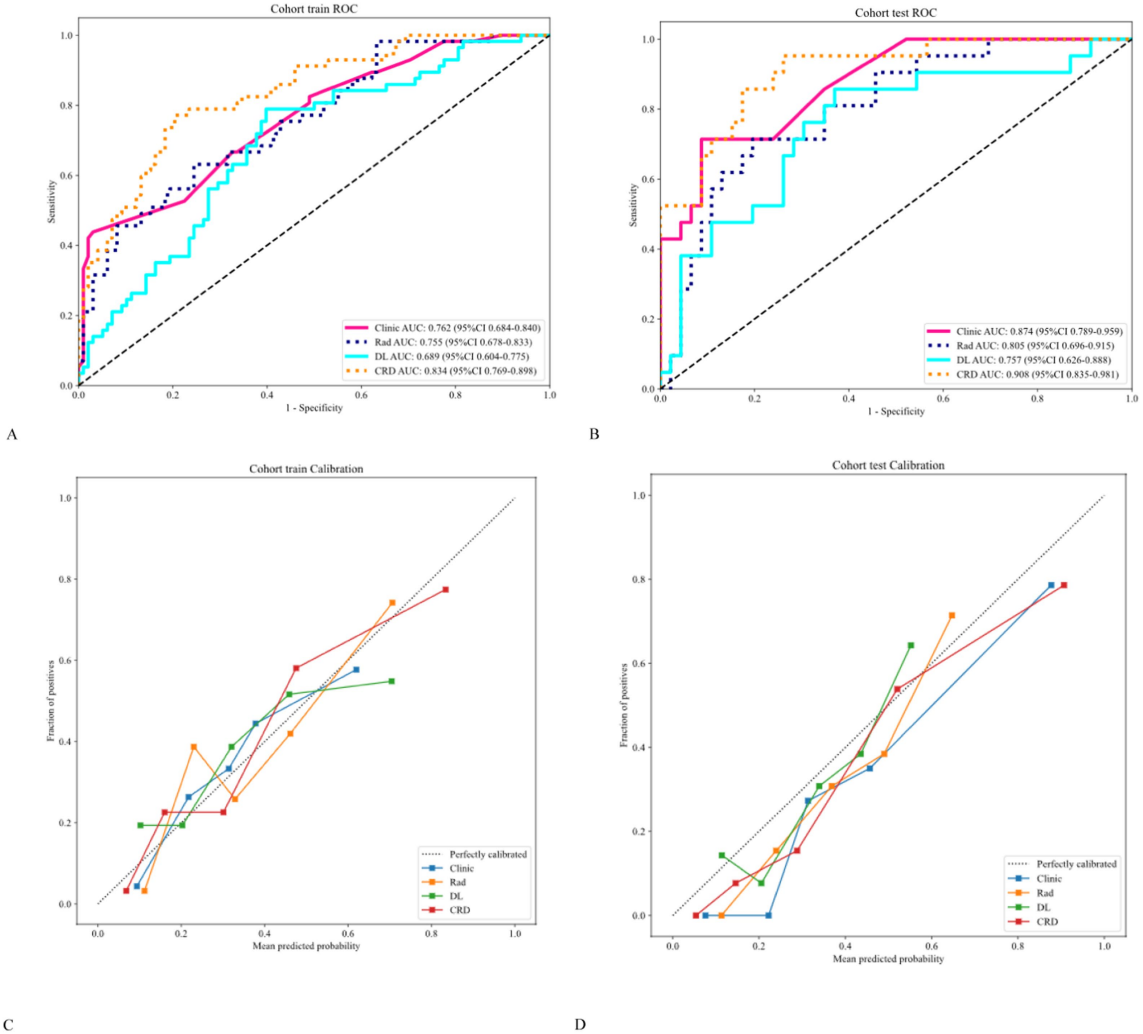
**(A,B)** Receiver operating characteristic curves showing that the CRD model exhibited significantly higher AUC values than the other methods in both cohorts. **(C,D)** Calibration curves showing that the CRD model exhibited exceptional consistency and calibration in predicting a poor prognosis for patients with AIS.

The AUC analysis of the specific indicators revealed varying degrees of performance across the different methods and cohorts (Figure 4). In the training cohort, the clinical, radiomics, deep learning, and CRD models achieved AUC values of 0.762, 0.755, 0.689, and 0.834, respectively (Table 3). In the validation cohort, the clinical model exhibited an AUC of 0.874, the radiomics model achieved an AUC of 0.805, the deep learning model attained an AUC of 0.757, and the CRD model again outperformed all other methods with an AUC of 0.908. These findings suggest that the CRD model exhibited the most consistent and robust performance in distinguishing between classes, with significantly superior AUC values compared to the other methods in both the training and validation cohorts.

**Table 3:** Predictive performance of different models to estimate the risk of poor prognosis.

Model	Accuracy	AUC	95% CI	Sensitivity	Specificity	PPV	NPV	Cohort
Clinic	0.774194	0.762084	0.6842–0.8399	0.421053	0.979592	0.923077	0.744186	Training
Radiomics	0.703226	0.75546	0.6776–0.8333	0.614035	0.755102	0.59322	0.770833	Training
Deep learning	0.664516	0.689402	0.6040–0.7748	0.77193	0.602041	0.53012	0.819444	Training
CRD	0.780645	0.833691	0.7691–0.8983	0.754386	0.795918	0.68254	0.847826	Training
Clinic	0.791045	0.874224	0.7894–0.9591	0.523809	0.913043	0.733333	0.807692	Validation
Radiomics	0.761194	0.805383	0.6956–0.9152	0.666667	0.804348	0.608696	0.840909	Validation
Deep learning	0.686567	0.756729	0.6258–0.8876	0.809524	0.630435	0.5	0.878788	Validation
CRD	0.791045	0.907867	0.8352–0.9805	0.904762	0.73913	0.612903	0.944444	Validation

**Figure 5:**
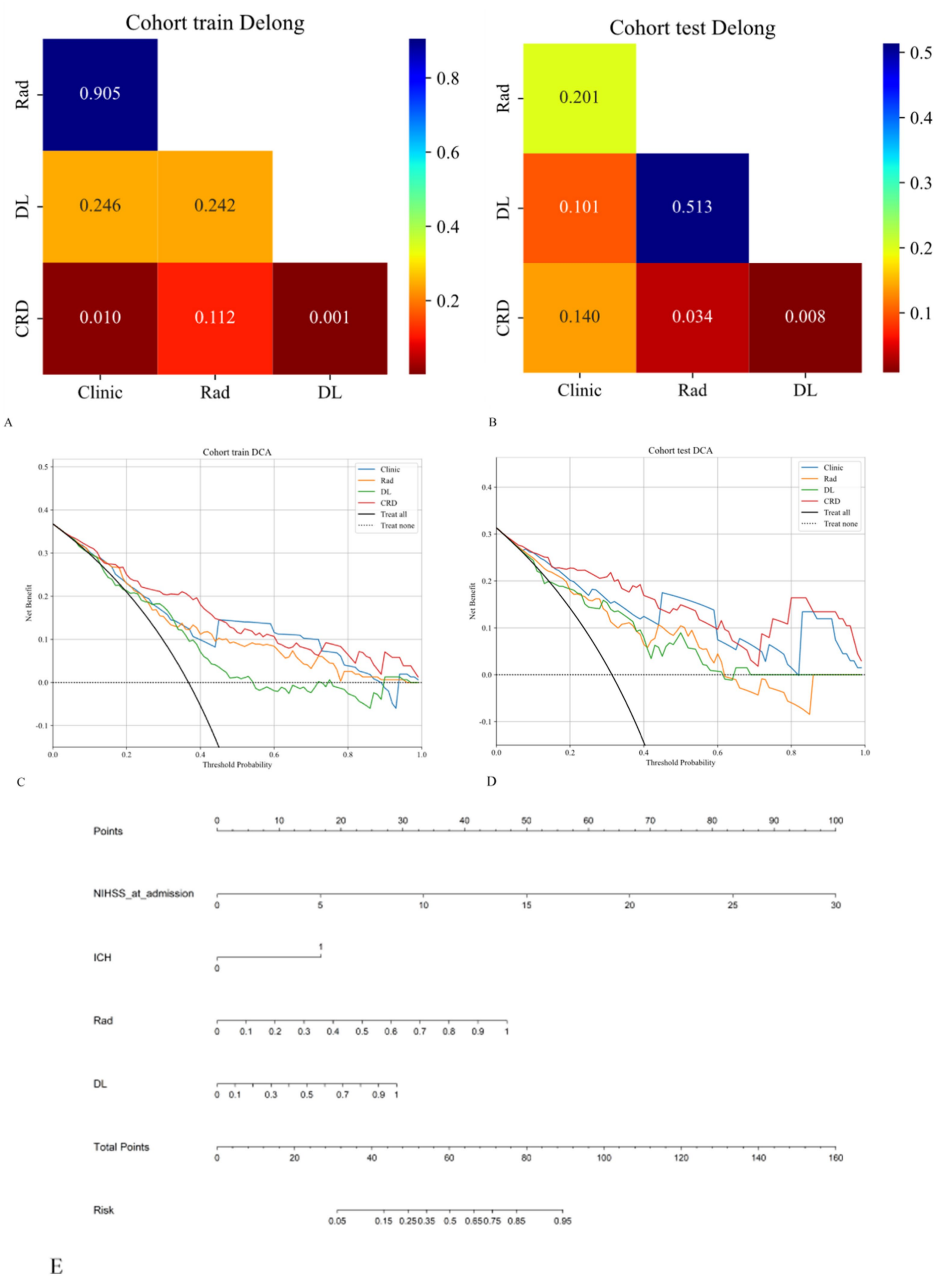
**(A,B)** The DeLong test was applied to both the training and validation cohorts to evaluate the statistical significance of the differences between the models. **(C,D)** DCA curves demonstrating that the CRD model offers the greatest net benefit compared to the clinical, radiomics, and deep learning models. **(E)** A nomogram was constructed for the CRD model based on the NIHSS score at admission, ICH, radiomics score, and deep learning score.

The calibration curve revealed that the CRD model demonstrated exceptional consistency and calibration in predicting poor stroke prognosis and actual results (Figure 4). The Hosmer–Lemeshow test showed that *P* was > 0.05, indicating that there was no significant difference between the predicted and true values. DeLong’s test indicated that in the training cohort, the CRD model outperformed both the clinical and deep learning models (*p* = 0.01 and *p* = 0.001, respectively). In the validation cohort, the CRD model surpassed the radiomics and deep learning models in terms of predictive performance (*p* = 0.01 and *p* = 0.008, respectively; Figure 5). Figure 5 also shows the DCA for the four models, with the CRD model achieving the highest net benefit compared with the radiomics, deep learning, and clinical models. Using the CRD model, a visual nomogram (Figure 5) was constructed to estimate the risk of a poor prognosis. As illustrated in the nomogram, the NIHSS score at admission was the most influential factor in the scoring system.

## 4 Discussion

AIS is a non-communicable disease that severely threatens public health and is characterized by high incidence, disability, recurrence, and economic burden. The lifetime risk of stroke is notably elevated among individuals aged ≥25 years in China, with the recurrence rate in the first year after the initial stroke ranging between 9.8 and 23.0% (9, 10). Recurrent strokes are associated with high rates of disability and mortality. Although mechanical thrombectomy in patients with acute stroke achieves a high recanalization rate, a significant proportion of patients still experience poor outcomes. Early prediction of the functional prognosis allows for timely intervention and rehabilitation, such as blood pressure and glucose control, individualized early anticoagulation and antiplatelet therapies, and neurocognitive rehabilitation, thereby enhancing the patient’s quality of life (11). Therefore, early prognostic evaluation is of great significance in guiding personalized clinical treatment strategies. Previous studies have shown that factors such as age, atrial fibrillation, and NIHSS scores are closely associated with stroke prognosis. However, the mechanisms underlying functional outcomes after mechanical thrombectomy for acute stroke are complex, and the prediction of stroke prognosis remains controversial (12).

In recent years, neuroimaging technologies have evolved from basic diagnostic tools to play more critical roles, particularly in guiding reperfusion therapy and predicting prognosis. Currently, AIS is primarily diagnosed using CT and MRI, with DWI and FLAIR sequences being particularly sensitive to ischemic stroke (13). The DWI sequence, as part of the first-line diagnostic approach for acute stroke, is considered the most accurate method for assessing infarct volume, and MRI may play a crucial role in predicting AIS recurrence. High-signal areas on DWI are typically indicative of the core infarct regions (14). Previous studies have suggested that the infarct volume in patients with acute stroke correlates closely with prognosis, with smaller infarct volumes before treatment often being associated with better outcomes. However, the manual evaluation of MRI images is inherently subjective, and the predictive capacity of traditional imaging parameters for stroke prognosis remains limited (15).

In recent years, radiomics has emerged as a prominent research area that provides multiparametric, morphological, and functional data. Radiomics transcends traditional medical imaging models based on morphology and semi-quantitative analysis by utilizing high-throughput feature extraction algorithms to quantitatively analyze imaging data (16). This approach allows for comprehensive exploration and analysis of the hidden information embedded within images, thereby optimizing the utility of imaging results and supporting personalized treatment strategies in clinical practice. Radiomics has demonstrated immense potential as an advanced technological tool in the field of oncology. This success can be attributed to the support provided by genomic projects and biomolecular research data, which have enabled researchers to apply radiomics to tumor imaging and extract valuable insights from it. In tumor imaging, the application of radiomics has expanded to include the prediction of tissue pathology, tumor grading, genetic mutations, patient survival rates, and therapeutic outcomes (17).

However, the application of radiomics is not limited to tumor imaging; any digital medical image can benefit from radiomic analysis. Inspired by the successful experiences in tumor imaging, researchers have begun applying these techniques to non-oncological diseases, including cerebral aneurysms, ischemic stroke, hemorrhagic stroke, cerebral arteriovenous malformations, and demyelinating diseases. MRI radiomics holds significant value in predicting the prognosis of patients with AIS who have undergone mechanical thrombectomy (18). Studies have shown that effective prognostic models can be developed by extracting features from DWI sequences and employing support vector machine classifiers (19). Additionally, radiomics can be used to analyze the source of AIS thrombosis, thereby guiding clinical decisions regarding thrombolytic or thrombectomy approaches. In one study focusing on the prognosis of patients with stroke undergoing mechanical thrombectomy, those with higher NIHSS scores at admission typically had a poorer prognosis. Using radiomics models, multiple features that were significantly correlated with AIS prognosis were identified, including first-order, shape, and texture features (20). Among these, the GLCM reflects the homogeneity and heterogeneity of lesions, indirectly revealing the potential impact of stroke-related changes in heterogeneity on patient prognosis. GLRLM, on the other hand, captures the directional and roughness aspects of the image texture, where directional textures may exhibit longer runs at specific angles. These features capture local heterogeneity and gray-level variations in images, providing a more accurate and comprehensive radiomic basis for patient prognostic evaluation (21).

Wang et al. (22) extracted 402 radiomics features from DWI sequences. Significant differences in age, infarct volume, baseline and 24-h NIHSS scores, and hemorrhagic status were observed between the groups with favorable and unfavorable functional outcomes. Eleven radiomic parameters were identified, showing strong predictive performance in both the training and validation cohorts, with AUCs of 0.69 (0.59–0.78) and 0.73 (0.63–0.82), respectively. A radiomic nomogram combining clinical features (age, hemorrhage, and 24-h NIHSS score) and radiomic features showed strong discriminatory power in the training cohort (AUC = 0.80; 95% confidence interval [CI] 0.75–0.86) and was validated in the validation cohort (AUC = 0.73; 95% CI 0.63–0.82). This study did not consider the location and size of the ischemic injury, which may have affected the results. Although radiomic features and clinical variables showed high specificity, their sensitivity was lower, likely because of the generally favorable outcomes in most patients.

Liu et al. (21) divided patients with AIS into recurrent and non-recurrent groups based on stroke recurrence within 1 year. From the 1,037 radiomic features extracted from the DWI images, 20 were selected for machine learning models. In the validation cohort, LightGBM exhibited the highest level of accuracy. The radiomic data yielded a sensitivity of 0.65, specificity of 0.671, and AUC of 0.647. The clinical data achieved a sensitivity of 0.7, specificity of 0.799, and AUC of 0.735. When combined, the data resulted in a sensitivity of 0.85, specificity of 0.805, and AUC of 0.789. The top factors of the LightGBM model included clinical indicators, such as hemoglobin, platelet-to-large platelet ratio, and age, along with radiomic features. However, the study used only 2D images, limiting the potential of 3D imaging, and may have overlooked certain clinical factors. Future research should expand the dimensionality of the clinical data.

Compared with traditional methods that solely analyze imaging data, radiomics enables a deeper exploration of image information, facilitating the transformation of images into higher-dimensional data (23). This not only enhances the accuracy of prognostic assessments but also provides stronger support for clinical treatment decisions. Radiomic features can reflect the gray-level distribution within images and the interrelationships between voxels, and quantify the heterogeneity within lesions that are invisible to the naked eye, thus aiding in the recognition and classification of diseases (24). Radiomics has already been employed in stroke-related research, such as identifying acute cerebral infarction lesions based on CT- or MRI-derived radiomic features, with MRI-based radiomic features being particularly useful for assisting with the early diagnosis of post-stroke cognitive impairment. Previous studies have predominantly utilized DWI sequences for image processing and data extraction (25). MRI offers superior tissue resolution, demonstrates exceptional sensitivity and specificity for diagnosing AIS, and has gained widespread clinical recognition. Multimodal MRI, which combines conventional and specialized sequences, reflects the pathophysiological changes in ischemic brain tissue. Its utility extends beyond diagnosis, offering insights into collateral circulation, hemodynamics, and molecular metabolism (26). This comprehensive approach allows for an integrated evaluation of the cerebral parenchyma, cerebrovascular conditions, and cerebral hemodynamics, thereby providing a precise reflection of the pathological and physiological state of patients with AIS, ultimately guiding the development of more personalized and accurate treatment strategies.

Deep learning is a pivotal branch within the broader field of machine learning. It emulates the learning process of the human brain through the construction of multilayered neural networks, thereby enabling comprehension and analysis of intricate data (27). Compared with traditional machine learning algorithms, deep learning models exhibit a superior capacity for representation learning and generalization, autonomous extraction of features from data, and the generation of higher-level abstract representations (28). The fundamental concept of deep learning is the iterative transformation of data features through successive layers of neural networks, effectively mapping data from a raw, low-level feature space to a more advanced, abstract feature space (29). In this process, each layer applies a non-linear transformation to the output of the preceding layer, thereby extracting increasingly abstract and meaningful features. This layered transformation enables deep-learning models to address increasingly complex and abstract tasks. The success of deep learning can be attributed to the availability of vast datasets, formidable computational power, and advanced algorithmic models. With the widespread proliferation of the Internet and the acceleration of the digitalization process, the volume of data available has increased exponentially. Such data provide rich training and testing samples, facilitating outstanding performances using deep learning models across diverse and complex scenarios. As computer hardware continues to evolve and parallel computing technologies advance, the training time of deep learning models will be significantly reduced, making deep learning more practical for real-world applications.

Deep learning is progressively transforming our understanding and practice of medicine. Owing to its robust capabilities in feature extraction and pattern recognition, deep learning technology has instigated revolutionary changes in various facets of medical practice, including diagnosis, treatment, and prognostic evaluation. For example, CNNs have been extensively applied for the automatic analysis of pulmonary CT images, aiding in the detection and diagnosis of diseases such as lung cancer. Moreover, deep learning models can segment and annotate medical images, facilitating more precise localization and measurement of pathological areas (1). In addition, deep learning has demonstrated immense potential for disease prediction and prevention. By analyzing and learning from large-scale medical datasets, deep learning models can identify the risk factors and early warning signals associated with specific diseases.

As medical technology continues to advance and digitalization accelerates, the volume of medical data is growing exponentially. In this context, deep learning, a powerful machine learning technique, has demonstrated enormous potential for processing and analyzing large-scale medical datasets (30). In particular, the application of deep learning in stroke diagnosis and treatment has attracted increasing attention. Traditional stroke diagnostic methods often rely heavily on the clinical experience and subjective judgment of healthcare providers. In contrast, deep learning can automatically extract and recognize complex features and patterns associated with stroke by learning from vast amounts of medical data, thereby enhancing the diagnostic accuracy and efficiency. An accurate assessment of the infarct core plays a pivotal role in predicting patient outcomes (31). Although CT is more convenient, it is not particularly sensitive to early infarction changes. To address this issue, Lu et al. (32) developed a deep learning model to identify early subtle AISs in non-contrast CT scans. Their CNN model effectively captured the deep image feature differences between the region of interest and normal tissue and successfully identified and localized lesions. Evaluation using the AUC, sensitivity, specificity, and accuracy metrics (with 95% CIs) showed that the diagnostic performance of the model significantly outperformed that of two experienced radiologists. After referencing the model, the diagnostic accuracy of the radiologists also showed marked improvement, with results highly consistent with the infarct lesion volumes obtained from DWI.

In this study, we developed a 2.5D CNN model based on ResNet101, utilizing residual structures to perform feature extraction from brain MRI images. Through transfer learning, the dataset features were converted into vectors, which were then fused through fully connected layers, to ultimately classify stroke prognosis. The model weights were saved when the highest accuracy was achieved in the validation cohort, and a deep learning model was subsequently constructed based on these parameters. This study introduces a 2.5D CNN model designed to extract brain MRI features and fuse multimodal information for the precise identification of stroke prognosis-related factors. While reducing the scale and parameter count of 3D convolution models, multimodal imaging is leveraged to ensure comprehensive feature extraction and accurate classification outcomes. This model does not require complex preprocessing of raw images, and the regions of interest in the images were validated using visualization techniques. The 2.5D CNN combines 2D and 3D convolutions, offering two distinct approaches for three-dimensional image segmentation: one based on 2D networks and the other on 3D networks. However, 2D network-based segmentation utilizes only in-slice information, whereas the 3D network approach may risk overfitting when computational resources are limited. The 2.5D method introduces interlayer information to enhance segmentation accuracy, considering the spatial information from adjacent layers. The fusion of multi-view perspectives and integration of adjacent layers as inputs, along with the incorporation of 3D features, significantly improved the model’s prediction results.

Magnetic resonance imaging, particularly quantitative susceptibility mapping and R2* relaxometry, plays a vital role in diagnosing AIS and elucidating its pathophysiology. These techniques can quantify iron concentration and myelin volume fraction, providing insights into the evolution of iron and myelination status in ischemic lesions. One study explored the relationship between iron deposition and myelination changes and neurological outcomes in patients with AIS. The results showed that patients with branch atheromatous disease (BAD) exhibited a higher susceptibility to changes, indicating increased iron deposition (33). Changes in NIHSS scores were significantly associated with changes in magnetic susceptibility values, but not with R2* values. Patients with increased iron and demyelination levels showed less improvement in neurological outcomes than those with decreased iron and remyelination levels. The BAD subtype, characterized by increased iron content and demyelination, was associated with worse neurological outcomes.

The ischemic penumbra, a region between irreversibly infarcted and normal brain tissue, is crucial in acute stroke treatment. Existing detection methods, such as ^15^O-positron emission tomography, are considered the gold standard, but are impractical in emergency settings. One study investigated the feasibility of using quantitative susceptibility mapping to estimate the oxygen extraction fraction for detecting the ischemic penumbra in patients with AIS (34). In 11 patients with a perfusion-core mismatch ratio ≥1.8, the volumes of increased oxygen extraction fraction (>51.5%) correlated positively with the ischemic penumbra volumes (*r* = 0.636, *p* = 0.035) and negatively with the 30-day change in NIHSS scores (*r* = −0.624, *p* = 0.041). The Dice similarity coefficient between the penumbra volumes analyzed using both the Dice similarity coefficient and oxygen extraction fraction methods was 0.724, indicating high consistency.

This study has several limitations that need to be addressed. First, it was a retrospective analysis with an insufficient sample size. Our analysis was based on a single-center study and lacked independent external validation, which restricted its generalizability. Second, the imaging data utilized in the study were obtained at the time of discharge, and the duration of clinical trial participation varied across cases, potentially limiting the predictive capability of the model in the early stages. Third, we did not perform a subgroup analysis of anterior and posterior circulation strokes. Given the substantial differences in infarction mechanisms and prognostic factors between these regions, such an analysis is crucial for uncovering the specific biological associations of a model.

## 5 Conclusion

The CRD model based on multimodal MRI demonstrated high diagnostic efficacy and reliability in predicting poor prognoses in patients with ischemic stroke. This approach holds considerable potential to assist clinicians in the risk assessment and decision-making processes for patients with AIS.

## Data Availability

The original contributions presented in the study are included in the article/Supplementary material, further inquiries can be directed to the corresponding author.

## Footnotes

^1^ http://www.itksnap.org/
