# Supplementary figures and images for: Prediction of prognosis in acute ischemic stroke after mechanical thrombectomy based on multimodal MRI radiomics and deep learning
Source: Front Neurol. 2025 Apr 30;16:1587347. doi: 10.3389/fneur.2025.1587347 (PMC12074947; doi:10.3389/fneur.2025.1587347)

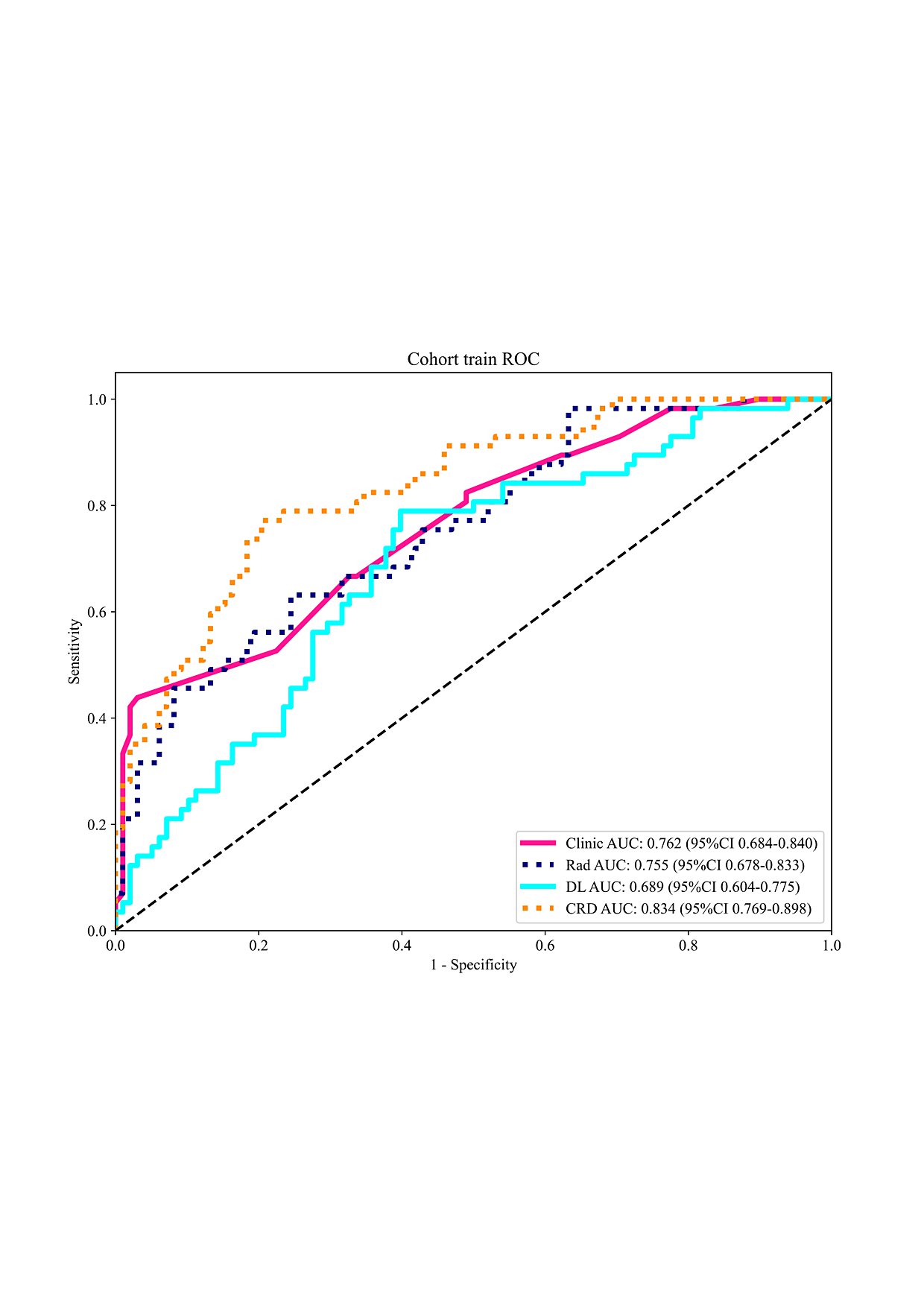

Supplement: Supplementary file 1 [file Image_1.JPEG]

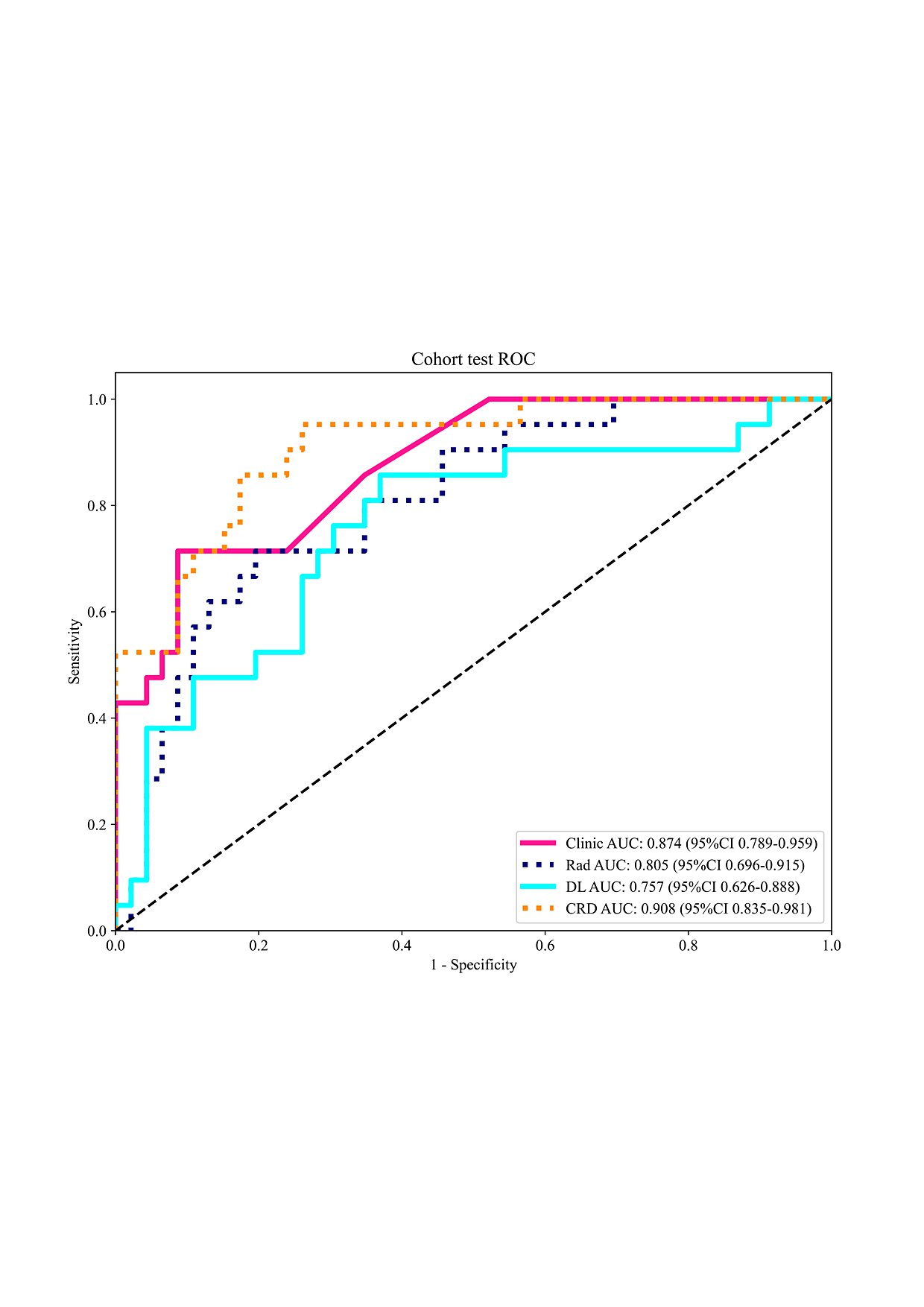

Supplement: Supplementary file 2 [file Image_2.JPEG]

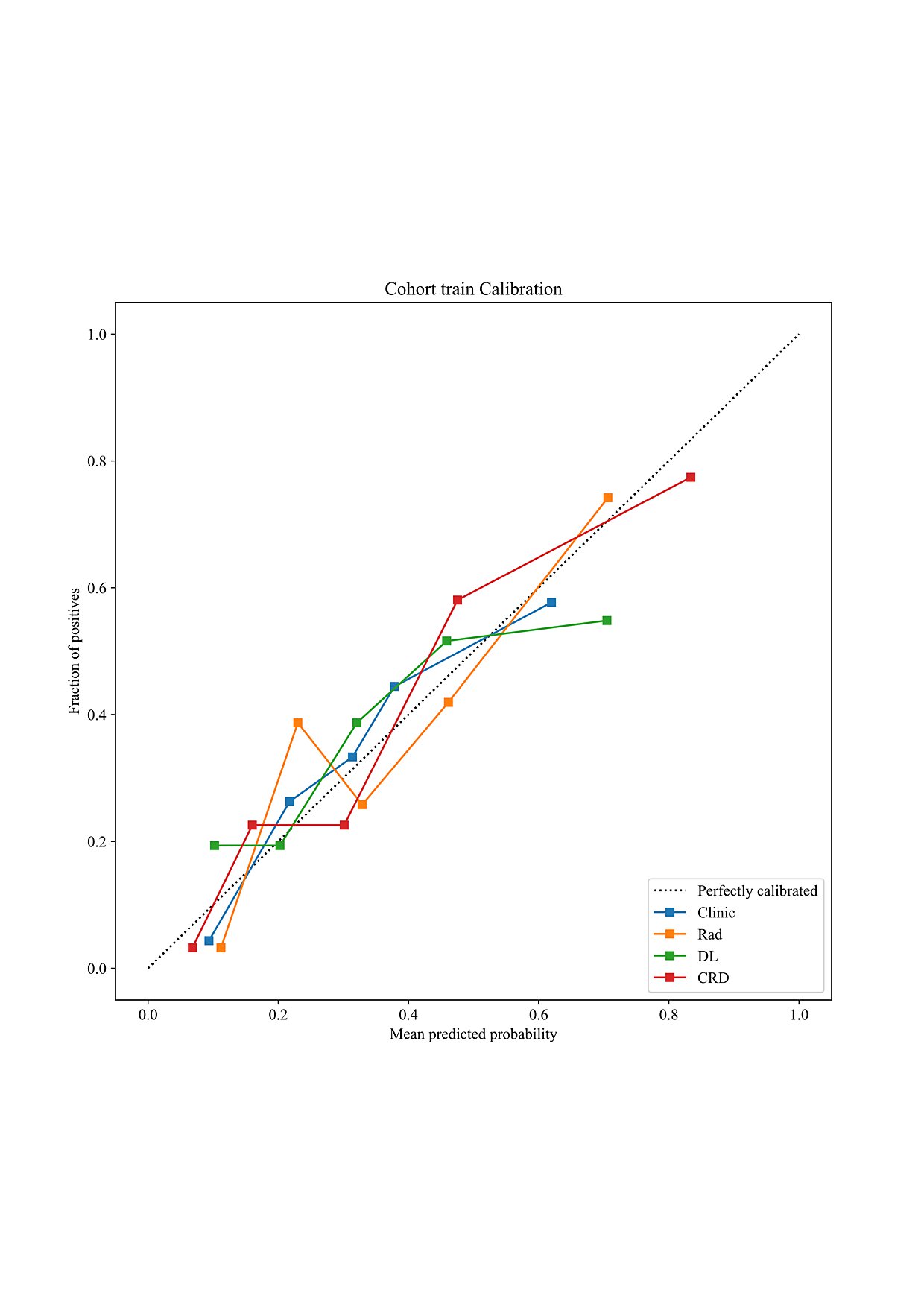

Supplement: Supplementary file 3 [file Image_3.JPEG]

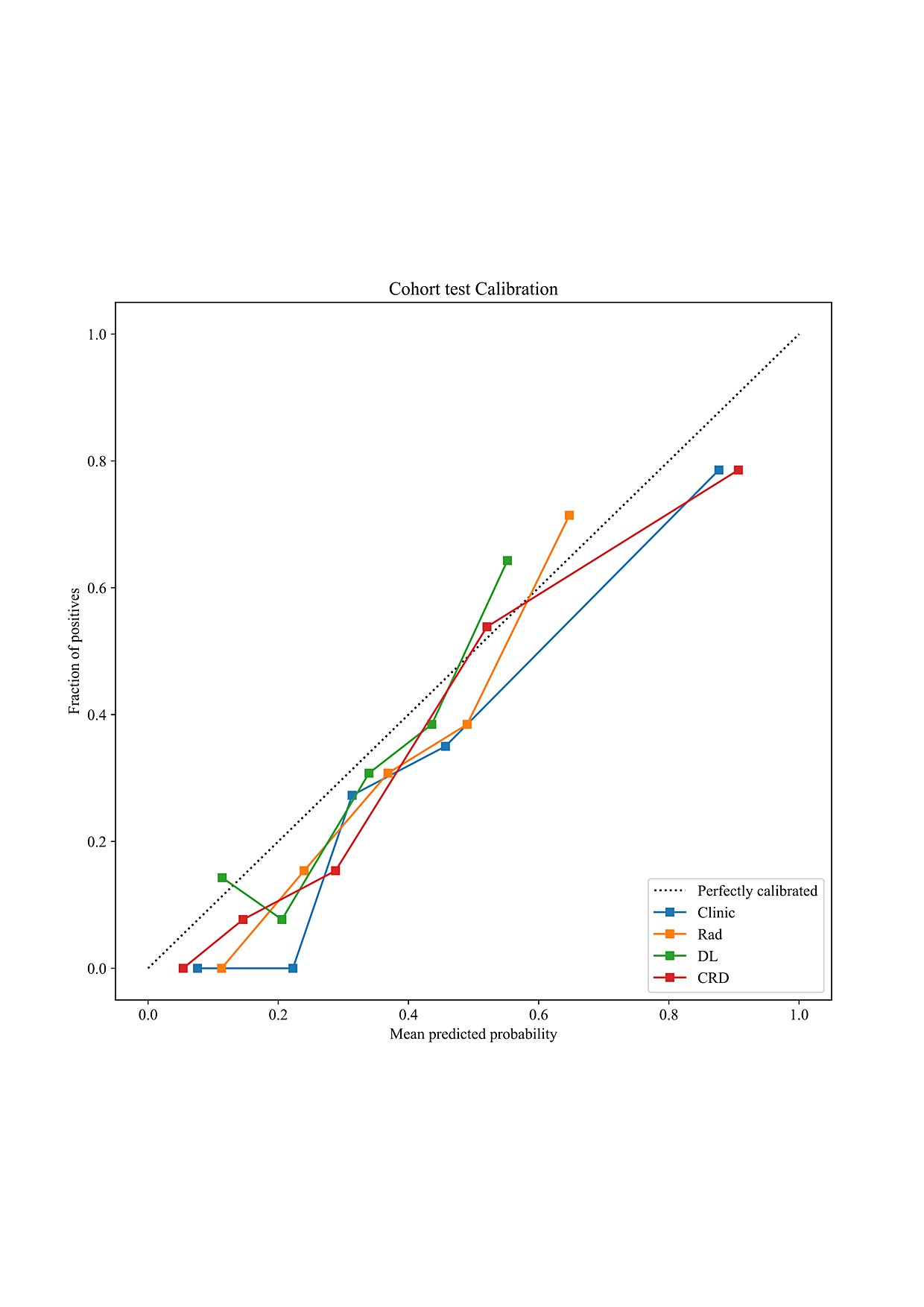

Supplement: Supplementary file 4 [file Image_4.JPEG]

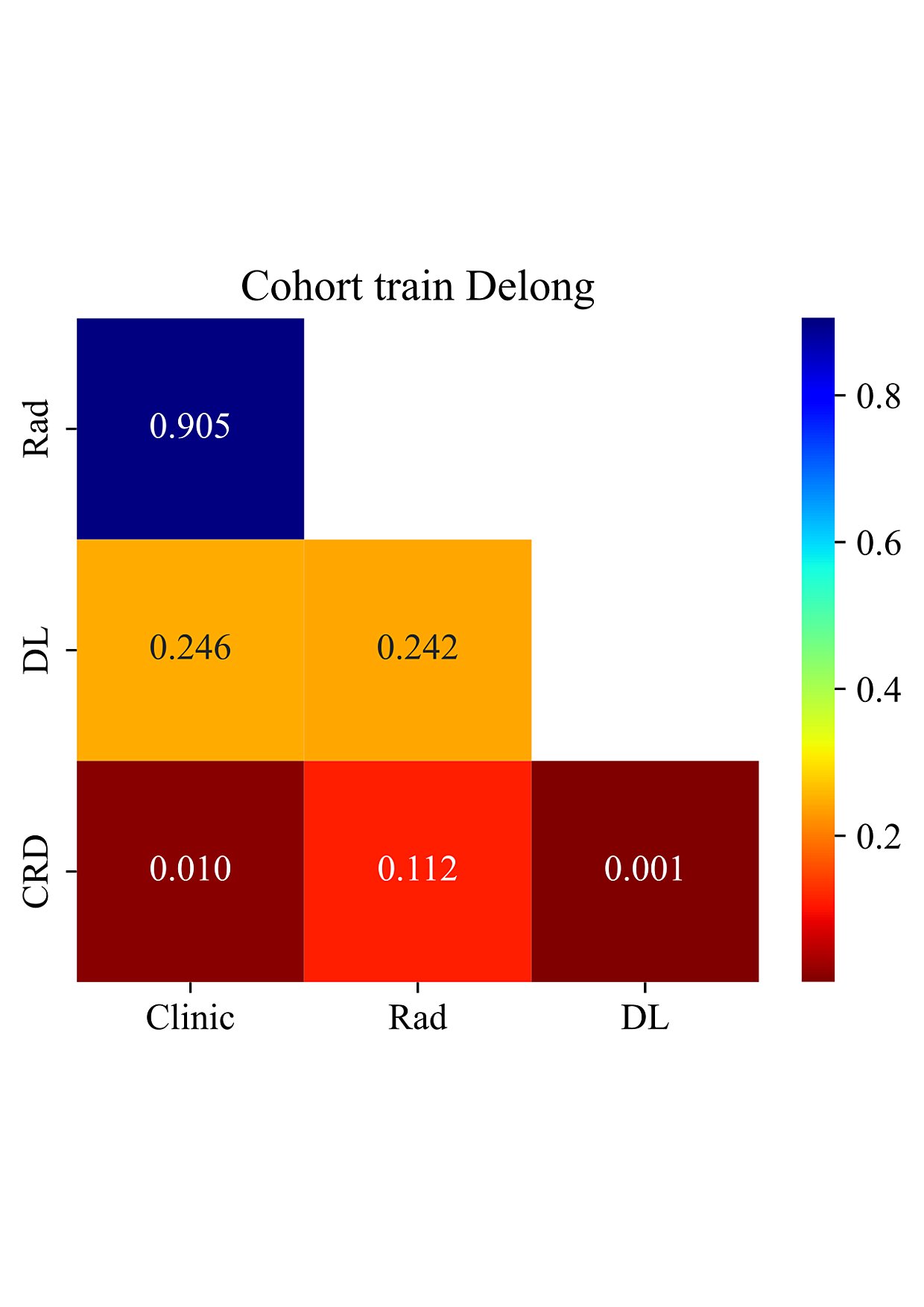

Supplement: Supplementary file 5 [file Image_5.JPEG]

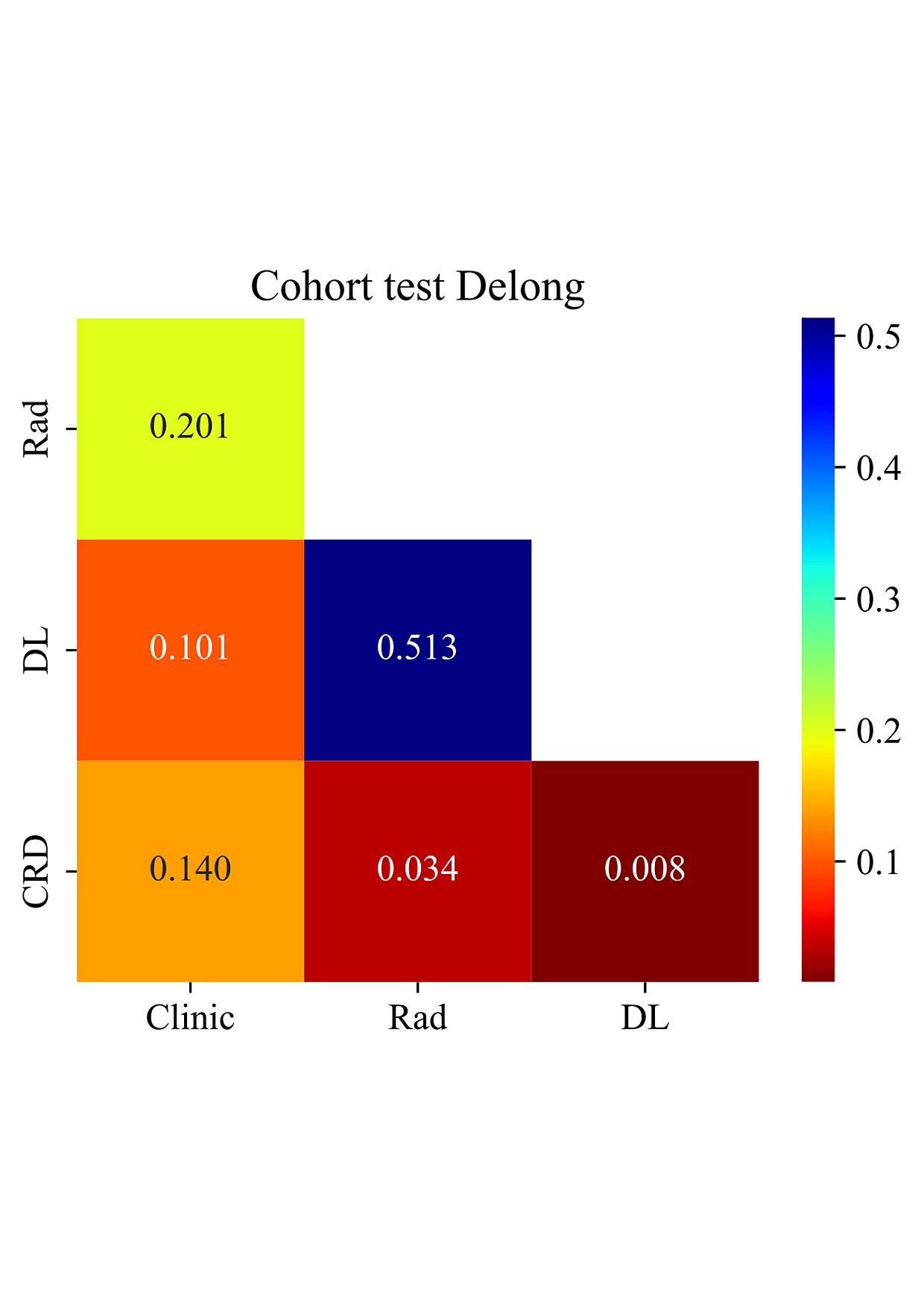

Supplement: Supplementary file 6 [file Image_6.JPEG]

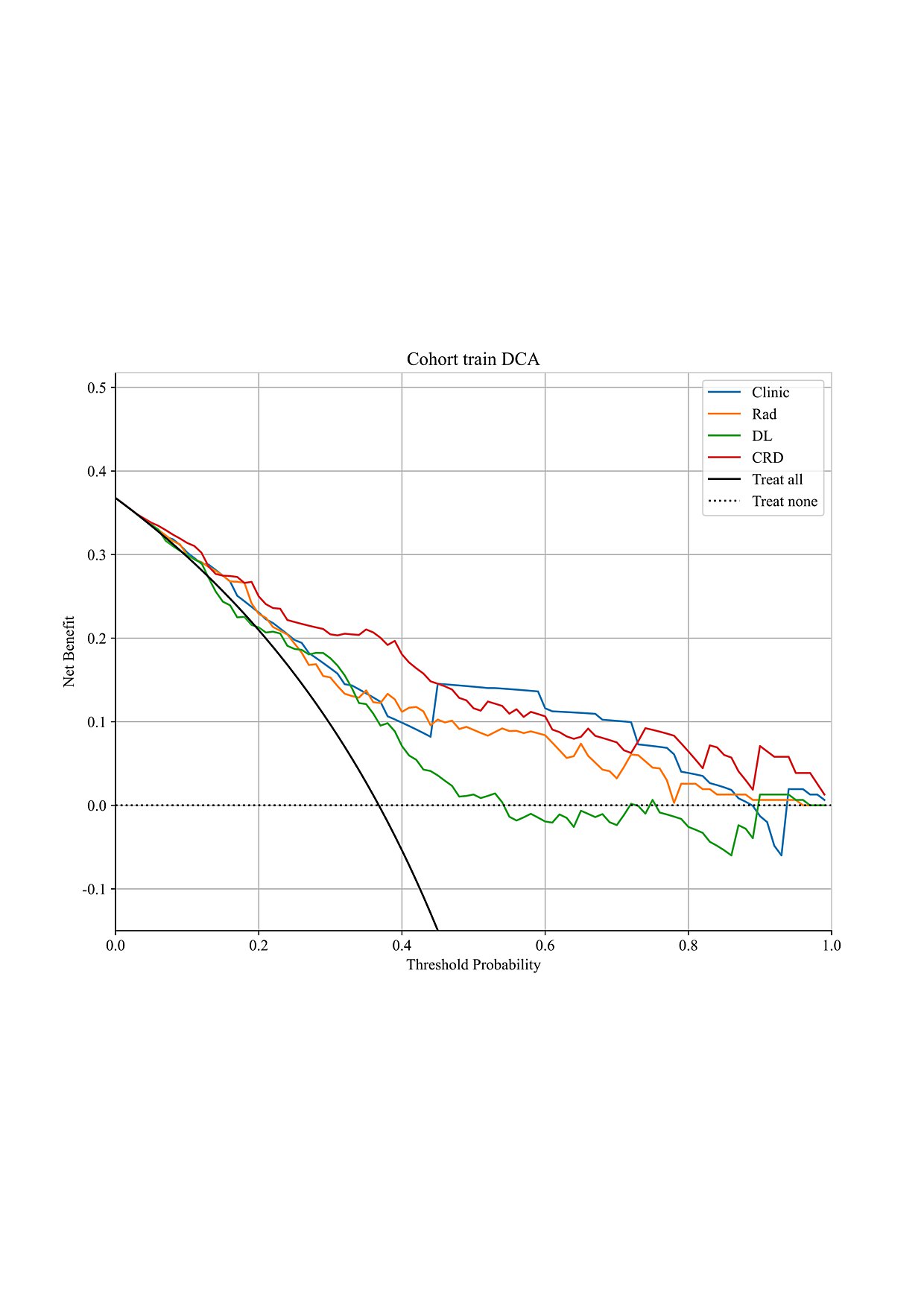

Supplement: Supplementary file 7 [file Image_7.JPEG]

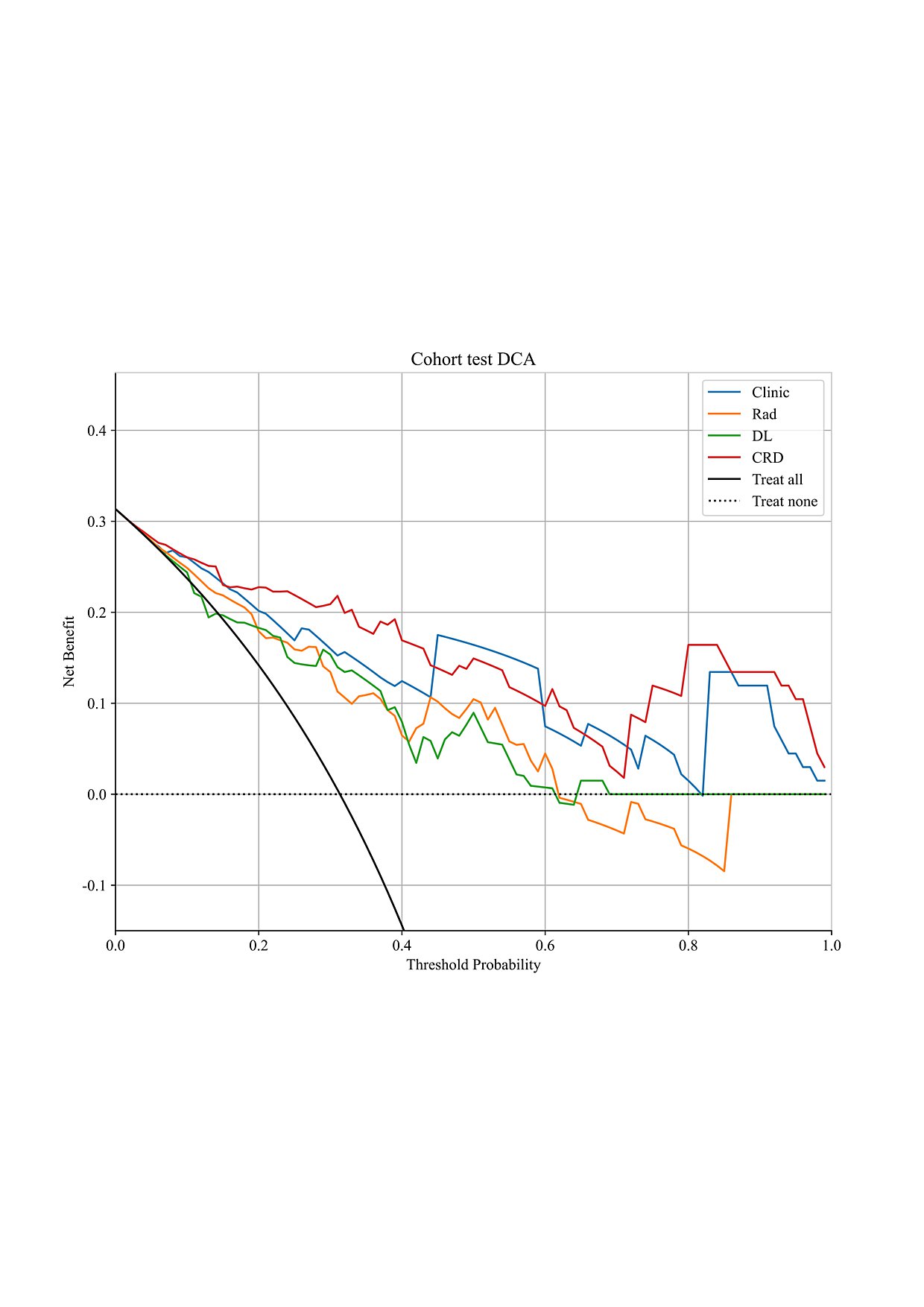

Supplement: Supplementary file 8 [file Image_8.JPEG]

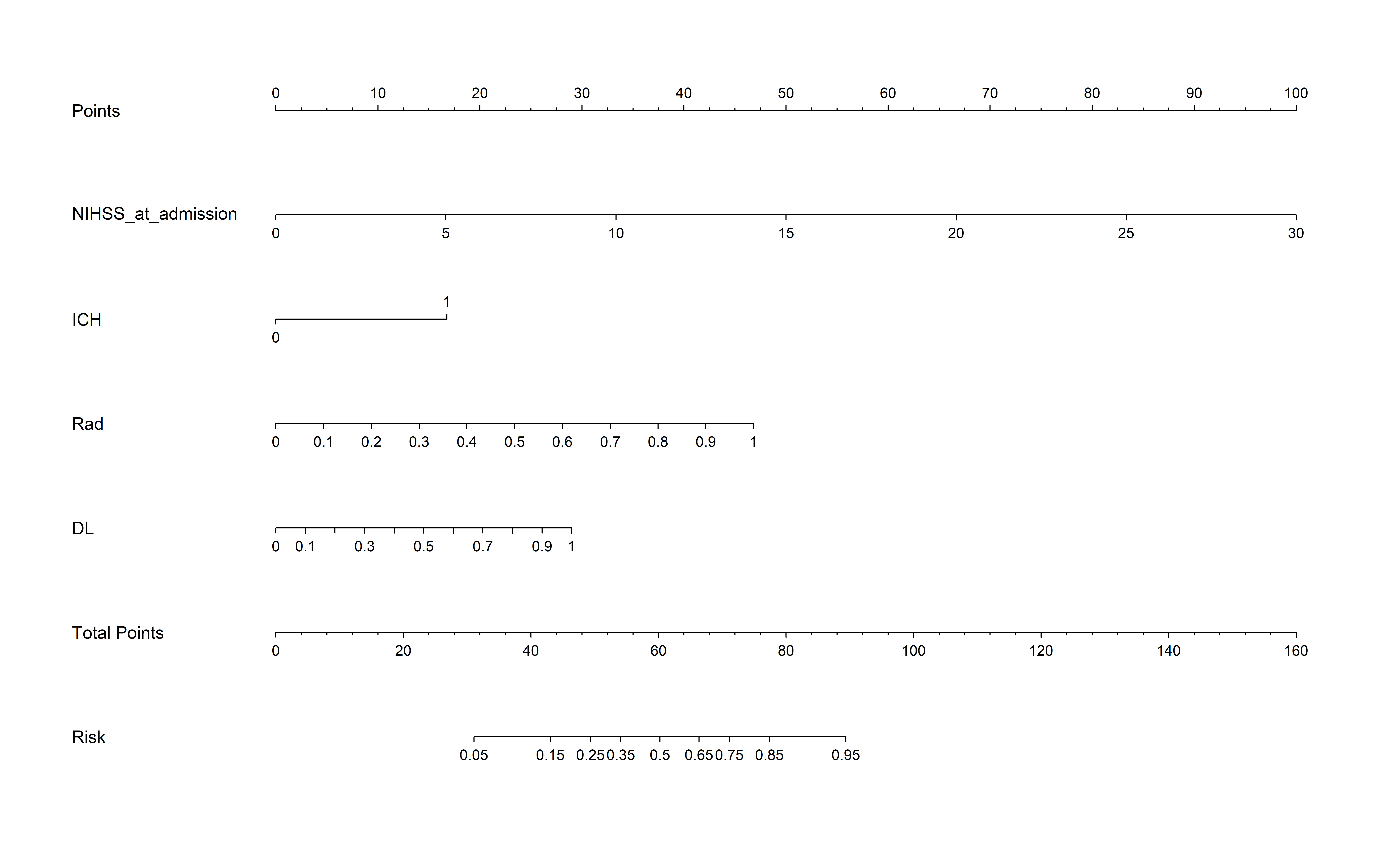

Supplement: Supplementary file 9 [file Image_9.JPEG]
